# Reflections on the Past 2½ Years and Inspiration for the Future

**DOI:** 10.31486/toj.22.5030

**Published:** 2022

**Authors:** Suma Jain

**Affiliations:** Department of Pulmonary and Critical Care, Ochsner Clinic Foundation, New Orleans, LA and The University of Queensland Medical School, Ochsner Clinical School, New Orleans, LA

*Editor's Note: Dr Suma Jain was one of several Ochsner board members invited to address the Ochsner Physicians Leadership Retreat on September 16, 2022. Her reflections on the past 2½ years left the audience spellbound. Her message powerfully resonated with all in attendance. This is an excellent message to help close out Ochsner's 80th anniversary—a look to the future while recognizing where we have come from. –R.A.*

I want to talk not about the last 80 years but the last 2½ years, which to many of you and especially to me feel like 80 years, or at least I feel like I’ve aged 80 years.

Sometimes, I think about where we were in March 2020. We had a lot of uncertainty, fear, and anxiety. We had no idea what was coming for us. In the words of Donald Rumsfeld, it was really the “unknown unknowns.”

In some ways, it was similar to a hurricane. In the beginning, it was a little exciting, watching the news, reading the reports and updates, monitoring the barometric pressures, except we couldn’t escape or evacuate. And then everything everywhere shut down—everywhere except at the hospitals. We had virtual school, virtual meetings, then virtual appointments, virtual galas, virtual happy hours, and virtually everything changed.

And we still had no idea what was ahead for us.

When asked in March 2020, I told people that it was going to be done by August! Talk about unknown unknowns. Just when we thought we might be out of the woods in the late spring of 2020, we had the July summer COVID surge and then hurricanes Marco and Laura, and then in October hurricanes Delta and Zeta, and then the COVID winter surge (we are still in 2020), and then in the middle of summer 2021 the Delta COVID surge and Hurricane Ida!

Oh, and then there was the Omicron surge of winter 2021.

I like to tell people that we here in the Gulf South handled COVID while recovering from 5 hurricanes in less than 1 year, whereas everyone else had COVID alone.

Also, to be clear, it was exciting until it wasn’t. Until it was exhausting, dehumanizing, and frustrating. It was almost enough for the strongest of us to give up. But COVID sometimes makes me think about Elisabeth Kübler-Ross's five stages of grief: denial, anger, bargaining, depression, and acceptance.

*Denial:* Those of you who were with me at the Red Tie Gala in March 2020 on a cruise ship with no windows and no masks, only days before our first patient came into the hospital. We were in denial.

*Anger:* At this virus for putting us in harm's way by wounding or killing our patients, friends, and colleagues. Initially anger for our patients and their families who were dying alone in the hospital, and then later, anger at some of our patients for not protecting themselves and others and denying us of our connections to each other.

*Bargaining:* We were bargaining with the disease. If we stay home, maybe we can be protected. If we wear a mask, maybe we can be protected. If we get vaccinated, maybe we can be protected.

*Depression*: At the loss of our lives both literally and figuratively, and sometimes the loss of our humanity.

And at the end, hopefully, *Acceptance*.

Just like the hurricanes, COVID did something else. It brought us all together. Especially in the beginning, we were all working together as one unit with one goal and one purpose: to take care of our patients and take care of each other. And that was so inspiring. This is where the Ochsner group practice flourished. I was on one night and got a call that one of the doctors on the 16th floor needed help. So I went up there, and it was one of my friends, a fellow Davidson alum, who is an orthopedic surgeon. After we took care of the patient, I asked him, “Why are you here? You are an orthopedic surgeon, and this is not what you do typically.” He said, “I felt like it was my duty. These people are my colleagues and my friends, and I needed to be here.”

I think about this support from my colleagues in the ED, internal medicine, cardiology, anesthesia, pediatrics, and surgery and all of you who volunteered to be in harm's way with us. That kind of commitment from my colleagues and our group practice is what gives us all the strength and inspiration to keep taking care of our patients and reminds us why we do what we do.

I think of all the support from my family across the country and the world, support from my neighbors and our friends who cooked us dinner and helped take care of our kids, and I think about the support from our patients and their messages of hope and prayer. I think about the support from hospital administrators who rounded at 6:00 AM with us, who joined clinical meetings to see what we needed, and who pumped gas and kept afloat both our finances and our spirits. And I think about all the people who were counting on us to care for them and their families and their communities and I am again reminded of why I do what I do.

And so now after 2½ years, I am reminded of “Invictus” by William Ernest Henley; we have emerged with our heads bloody, but unbowed, well, maybe a little bowed.

In some ways, trials like the pandemic and the hurricanes may have helped us reflect on what's most important to us and why we do what we do. Something we may not have done otherwise. So, we hope this meeting today will help you to reflect, find acceptance, reconnect with your purpose, and inspire you for the future.

